# Biodiversity explains maximum variation in productivity under experimental warming, nitrogen addition, and grazing in mountain grasslands

**DOI:** 10.1002/ece3.4483

**Published:** 2018-09-05

**Authors:** Jiajia Liu, Detuan Liu, Kun Xu, Lian‐ming Gao, Xue‐jun Ge, Kevin S. Burgess, Marc W. Cadotte

**Affiliations:** ^1^ Key Laboratory of Plant Resources Conservation and Sustainable Utilization South China Botanical Garden The Chinese Academy of Sciences Guangdong China; ^2^ Yunnan Key Laboratory for Integrative Conservation of Plant Species with Extremely Small Populations Kunming Institute of Botany Chinese Academy of Sciences Kunming Yunnan China; ^3^ Lijiang Forest Ecosystem Research Station Kunming Institute of Botany Chinese Academy of Sciences Kunming China; ^4^ Key Laboratory for Plant Diversity and Biogeography of East Asia Kunming Institute of Botany Chinese Academy of Sciences Kunming China; ^5^ Department of Biology Columbus State University University System of Georgia Columbus Georgia USA; ^6^ Department of Biological Sciences University of Toronto‐Scarborough Toronto Ontario Canada; ^7^ Ecology and Evolutionary Biology University of Toronto Toronto Ontario Canada

**Keywords:** experimental warming, functional diversity, grazing, multimodel inference, nitrogen addition, phylogenetic diversity

## Abstract

Anthropogenic global warming, nitrogen addition, and overgrazing alter plant communities and threaten plant biodiversity, potentially impacting community productivity, especially in sensitive mountain grassland ecosystems. However, it still remains unknown whether the relationship between plant biodiversity and community productivity varies across different anthropogenic influences, and especially how changes in multiple biodiversity facets drive these impacts on productivity. Here, we measured different facets of biodiversity including functional and phylogenetic richness and evenness in mountain grasslands along an environmental gradient of elevation in Yulong Mountain, Yunnan, China. We combined biodiversity metrics in a series of linear mixed‐effect models to determine the most parsimonious predictors for productivity, which was estimated by aboveground biomass in community. We examined how biodiversity–productivity relationships were affected by experimental warming, nitrogen addition, and livestock‐grazing. Species richness, phylogenetic diversity, and single functional traits (leaf nitrogen content, mg/g) represented the most parsimonious combination in these scenarios, supporting a consensus that single‐biodiversity metrics alone cannot fully explain ecosystem function. The biodiversity–productivity relationships were positive and strong, but the effects of treatment on biodiversity–productivity relationship were negligible. Our findings indicate that the strong biodiversity–productivity relationships are consistent in various anthropogenic drivers of environmental change.

## 1 INTRODUCTION

Anthropogenic impacts such as increasing temperature, higher nitrogen addition, and overgrazing all conspire to cause rapid declines in plant biodiversity worldwide, especially in mountain grassland ecosystems, which naturally elicits concern about the consequences for the maintenance of ecosystem functioning (Chapin et al., 2000; Cingolani, Noy‐Meir, & Diaz, 2005; Roth, Kohli, Rihm, & Achermann, 2013; Urban, 2015). The relationship between plant biodiversity and ecosystem function has been a major research topic in ecology for several decades, and while there is general empirical support for a positive effect of biodiversity on function from manipulative experiments (Balvanera et al., 2006; Cardinale et al., 2006; Tilman, Isbell, & Cowles, 2014), there is a lack of clarity about how anthropogenic changes in plant biodiversity might affect biomass production in more natural systems (Zavaleta & Hulvey, 2007). Inconsistent biodiversity effects on productivity could result from how biodiversity is measured, the confounding effect of environmental heterogeneity, and the nature of the anthropogenic impacts.

One reason might be that traditional biodiversity measures, like species richness, do not sufficiently capture the critical processes such as resource complementarity and interspecific interactions that are responsible for ecosystem function, which might be better reflected in relevant traits or evolutionary histories of species in a community (Lavorel & Garnier, 2002; Partel, Laanisto, & Zobel, 2007). Recently, a number of studies have shown that measures based on phylogenetic or single or multiple functional traits appear to be superior to species richness in explaining variation in productivity of plant communities (Cadotte, 2013; Cadotte, Cavender‐Bares, Tilman, & Oakley, 2009; Flynn, Mirotchnick, Jain, Palmer, & Naeem, 2011; Liu, Zhang et al., 2015), and further supply direct links to the mechanisms controlling productivity (Cadotte, 2017). In addition to this, some studies (Liu, Zhang et al., 2015) found that statistical models that combined different biodiversity facets maximally explained the effects of biodiversity loss on ecosystem functioning or services. For example, Liu, Zhang et al. (2015) found that multivariate functional diversity was the single predictor that consistently outperformed other single‐biodiversity measures in explaining variation in productivity, but phylogenetic diversity and community‐level plant height combined to explain maximum variation. However, beyond biodiversity facets that represent species‐level differences, intraspecific variation is critically important to fully capture the diversity of plant communities (Albert et al., 2012). Ali and Mattsson (2017) evaluated the relative power of intraspecific and interspecific tree size variation and found that intraspecific variation better explained variation in aboveground biomass.

Although biodiversity is a major determinant of ecosystem productivity, the estimation of the biodiversity effect might be confounded by environmental factors and potential drivers of environmental change such as elevated temperature, nitrogen addition, and herbivory (Fridley, 2002; Hooper et al., 2005; Seabloom et al., 2017; Steudel et al., 2012; Tilman, Reich, & Isbell, 2012; Tilman et al., 2014). Thus, it is important to disentangle the relative importance of biodiversity relative to other drivers along an environmental gradient for inferring the consistent effects of biodiversity on the primary productivity of ecosystems. The majority of research on biodiversity effects on ecosystem function has been in experimentally assembled communities, and these studies generally support a positive relationship between biodiversity and ecosystem functioning (Hector et al., 1999; Liu, Zhang et al., 2015; Tilman et al., 2001). In contrast, biodiversity levels produced by an environmental gradient such as elevation might reveal different response of ecosystem productivity (Gough, Grace, & Taylor, 1994). Hence, the direct relevance of these experiments for estimating the impacts of realistic biodiversity loss due to environmental changes on ecosystem functioning remains controversial (Hector et al., 2007; Jiang, Wan, & Li, 2009).

To address the biodiversity–productivity relationships of natural communities under different anthropogenic impacts, we developed a fenced warming‐fertilizing experiment in mountain wetlands along an elevation gradient on Yulong Mountain, Yunnan, China. We employed open‐topped, passive warming chambers and urea fertilizer to simulate the projected global warming and nitrogen addition, respectively. We used a multimodel comparative approach to assess the relative contribution of single and various combinations of multivariate biodiversity indices, both with and without intraspecific variation, to predict the variance in biomass production after accounting for potential confounding factors including local environmental heterogeneity, warming, fertilizing, and grazing. We aimed to answer the following questions: (a) Does phylogenetic and functional diversity outperform traditional richness and evenness regardless of environmental heterogeneity and anthropogenic impacts? (b) Does incorporating intraspecific trait variability enhance the explanatory power of functional diversity? (c) Are biodiversity–productivity relationships comparable in experimental warming, nitrogen addition, and grazing along environmental gradient of elevation in mountain grasslands?

## 2 MATERIALS AND METHODS

### 2.1 Study sites and experimental design

We established eighteen study sites in south‐facing wetlands of regular topology of Yulong Mountain (100°10′E, 27°00′N) along an environmental gradient of elevation (2,700, 3,200, and 3,400m) within the Lijiang Alpine Botanical Garden of the Kunming Institute of Botany, Chinese Academy of Sciences in Lijiang, Yunnan Province, China. Yulong Mountain has the mean annual temperature of 12.8°C and the annual rainfall is 935 mm, which is mainly distributed from July to October with distinct dry and rainy seasons (Luo et al., 2016). Plant communities of wetlands have obvious species turnover along the elevation gradient with the dominance of the genera *Isachne, Juncus* at the lowest elevation, the genera *Ligularia*,* Agrostis* at the middle elevation, and the genus *Agrostis* at the highest elevation. All three wetlands have long livestock‐grazing histories, and each supports different types of livestock where sheep and horses graze at the lowest elevation, scalpers, and yaks graze at the middle and highest elevation, respectively.

**Figure 1:**
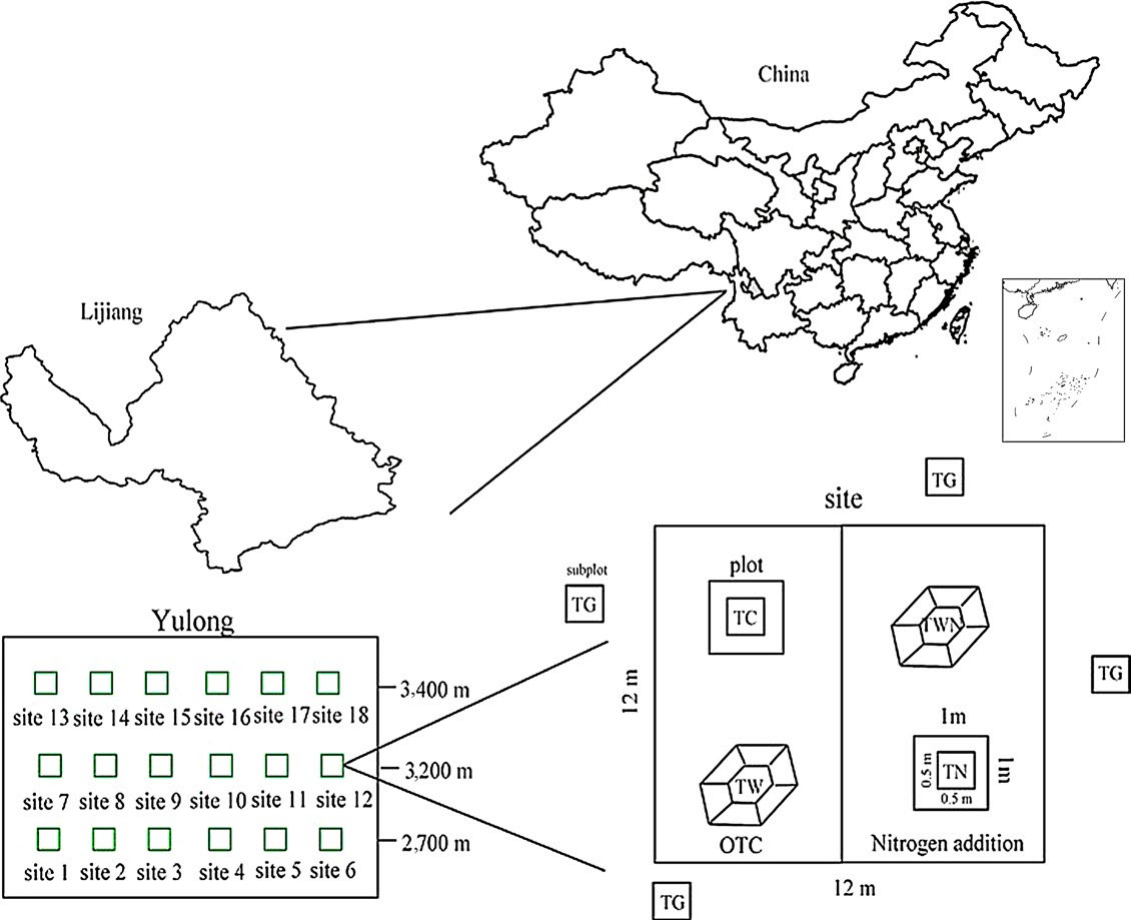
Map of the study sites on Yulong Mountain, Lijiang, Yunnan Province, China, and the plot design. Shown are treatments: *T*_C_ = control, *T*_W_ = warming, *T*_N_ = nitrogen addition, *T*_WN_ = warming and nitrogen addition, and *T*_G_ = livestock‐grazing

We established six 12 × 12 m permanent fenced sites randomly distributed in wetlands within each elevation in May 2015 (Figure 1). Within each permanent site, we conducted a complete randomized block factorial experiment with each block of size 5 × 5 m. There were two factors of both experimental warming and nitrogen addition in each block and two levels for each factor. In both fertilized blocks, we applied urea fertilizer annually at the beginning of the rainy season approximately the end of May at a rate of 5 g m^−2^ year^−1^. In both warmed blocks, we applied open top chambers (OTCs), commonly employed devices to study the effects of climate warming on ecosystems (Marion et al., 1997). Here, our open top chambers were octahedral frames made of angle iron, 1.5 m maximum diameter, and 45 cm height. Six sides of each open top chamber were fastened to transparent 1.5‐mm‐thick hard plastic with adjacent edges of two plastic pieces attached with adhesive. We regularly arranged two open top chambers and two corresponding plots of the similar area in four blocks with at least 3 m between the nearest edges of adjacent plots. Furthermore, we randomly positioned 3–4 plots around each permanent site with total of 20 grazed plots. Hence, there were five treatments (*T*_C_ = control, *T*_W_ = warming, *T*_N_ = nitrogen addition, *T*_WN_ = combination of warming and nitrogen addition, and *T*_G_ = livestock‐grazing; Figure 1).

We recorded species richness and their abundance in a rectangular subplot of 0.5 × 0.5 m from the center of each plot at the peak of the growing season in August 2016 (Figure 1). We then harvested all the stems of each species in each subplot at ground level, dried, and weighed them to 0.1 mg to estimate biomass production (productivity).

### 2.2 Environmental data

**Figure A1:**
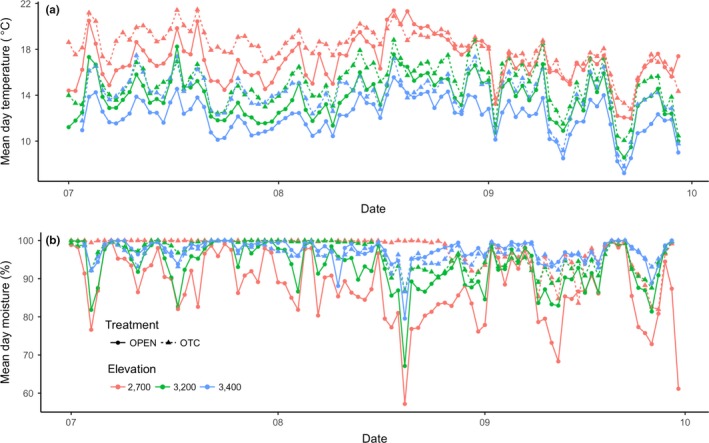
(a) Cumulative rainfall, (b) mean air temperature within the open top chamber (OTC) and control subplots, and (c) mean moisture within the open top chamber (OTC) and control subplots at the field sites (elevation: 2,700, 3,200, and 3,400) in the growing season (July–October) in 2016

After cutting the stems to ground level, we collected soil core samples from three random locations in each subplot with a cylindrical soil auger (5 cm inner diameter, 15 cm length). We combined the three replicates from the same depth for each subplot as a single composite sample, dried it in the shade, and filtered it using a 2‐mm sieve for stoichiometric analysis. We measured soil pH, concentration of nitrogen (N), phosphorus (P), and carbon (C) following the standard protocols (Sparks et al., 1996). Besides soil resources, we also collected climatic data for rainfall, air temperature, and air moisture using HOBO RG3‐M, HOBO Pro v2, respectively (Onset Computer Corporation, Bourne, MA, USA) from July to October in 2016. For each elevation, we placed one HOBO RG3‐M and two HOBO Pro v2, of which one was positioned inside an open top chamber and the other one was positioned in a control plot. We showed the detailed distributions of temperature and moisture during the experimental interval in Figure A1.

### 2.3 Plant traits and community phylogeny

We measured five plant traits including plant height (*H*, cm), leaf carbon content (LC, mg/g), leaf nitrogen content (LN, mg/g), leaf phosphorus content (LP, mg/g), and specific leaf area (SLA, cm^2^/g). These plant traits might reflect fundamental resource complementarity and interactions among co‐occurring species (Weiher et al., 1999; Wright et al., 2004). We recorded plant height of maximum five randomly selected individuals from each species in each subplot. We calculated the maximum of plant height for each species per plot for intraspecific variability among plots. We scanned at least 1 mature leaf of randomly selected five individuals per species in each subplot using an Epson‐V200 scanner. We then measured leaf area with image analysis software (ImageJ; http://rsb.info.nih.gov/ij). We weighed the leaves after dried to a constant weight at 60°C to 0.1 mg and calculated the specific leaf area as the ratio of leaf dry mass to leaf area. We pooled the leaves from different individuals of the same species and measured leaf carbon, nitrogen, and phosphorus content. For the missing traits data due to rare species, we substitute the average of the same traits of the same species or same genus within the same treatment.

We constructed the phylogeny for the 105 species recorded in our study using *rbcL* + *matK* regions of the chloroplast genome. The detailed descriptions of DNA extraction, amplification, and sequencing are provided in Liu, Yan et al. (2015). Here, we briefly described the inference method of phylogenetic reconstruction. We aligned the *rbcL* and *matK* sequences using MAFFT (Katoh & Standley, 2013) and concatenated *matK* to the *rbcL* to form a super matrix. We used the sequences from the same genus in BOLD as the substitutes for the missing sequences in 27 of the species. For each gene, we selected top‐ranked maximum‐likelihood model of nucleotide substitution using Akaike's information criterion, as implemented in the function modelTest in the *phangorn* library (Schliep, 2011) in R (R Core Team, 2016). Then, we estimated a maximum‐likelihood phylogeny using PhyML 3.0 with the starting‐tree estimated from the BioNJ (Guindon et al., 2010). We chose one representative of early diverging angiosperm lineage *Amborella trichopoda* as the root of phylogeny and then used a semiparametric rate‐smoothing method to transform the phylogeny to an ultrametric tree using the chronopl function with parameter value 1,000 in the R *ape* library (Paradis, Claude, & Strimmer, 2004).

### 2.4 Measures of biodiversity

Using species composition and number of individuals, we calculated traditional species richness (*S*) and Shannon's evenness index (*H′*) for each subplot. We also calculated a suite of single and multivariate functional diversity metrics based on plant traits, as well as phylogenetic diversity metrics using the maximum‐likelihood phylogeny. We listed the detailed descriptions of the measures of biodiversity in Table A1. Here, we give a brief description of important functional and phylogenetic metrics. To assess the potential effect of intraspecific trait variability, we averaged the traits for each species across all subplots in the study as its “fixed” traits and averaged the traits for each species in a given subplot as its “specific” traits. We then calculated a number of functional diversity metrics including single community‐level plant traits and multivariate functional diversity metrics for each subplot using both “fixed” and “specific” traits (Leps, de Bello, Smilauer, & Dolezal, 2011). Here, multivariate functional diversity metrics included Rao's quadratic entropy (RaoQ), which measures abundance‐weighted distances based on multiple traits (Botta‐Dukat, 2005) and functional richness (FRic), which measures the volume of the functional space occupied by the community (Villeger, Mason, & Mouillot, 2008). For the measures of phylogenetic diversity, we calculated the imbalance of abundances at higher clades (IAC), which encapsulates the distribution of individuals across the nodes in the phylogeny (Cadotte et al., 2010) and the abundance‐weighted mean nearest taxon distance in an assemblage (MNND; Cadotte et al., 2010).

### 2.5 General linear mixed‐effect models

We constructed a series of general linear mixed‐effect models to determine the most parsimonious relationships between productivity and the various measures of biodiversity, treatment, and local environmental factors including soil resources. We assumed that various measures of biodiversity, experimental treatments, and soil resources as fixed factors, whereas elevation, treatment, and plot were treated as hierarchical random factors. Here, the use of a normal distribution of model residuals was validated based on the normalized scores of standardized residual deviance (*Q–Q* plots). To evaluate model support, we used Akaike's information criterion corrected for small sample sizes (AIC_c_; Burnham & Anderson, 2002, 2004). We also used the marginal *R* ^2^ values of the models (*R*_m_^2^) as a measure of the model's goodness of fit (Nakagawa & Schielzeth, 2013).

To search for the most parsimonious models explaining patterns of biomass production, we firstly removed redundant predictors associated with phylogenetic, functional diversity metrics. We selected the relatively better‐ranked single‐biodiversity metric models in both phylogenetic and functional diversity metrics. Meanwhile, to testify whether experimental treatments affect biodiversity–productivity relationships, we regressed biomass production against each biodiversity metric with the addition or multiplication of treatment and compared the explanatory ability of these models using Akaike's information criterion weights. The detailed single‐biodiversity model ranking is listed in Table A2 and the biodiversity metrics we used in the following model construction are listed in Table 1.

**Table 1:** Measures of biodiversity for general multivariate linear mixed‐effect models

Biodiversity measure	Description	References
IAC	Imbalance of abundances among clades: measures the deviation in abundance distribution among internal splits from a null	Cadotte et al. (2010)
*H*_max_	Community‐level mean of plot‐specific maximum plant height values	Leps et al. (2011)
*LN*	Community‐level mean of mean leaf nitrogen content value for individual species used for all plots where the species is found	Leps et al. (2011)
*S*	Realized species richness of plot	Tilman, Wedin, and Knops (1996)
MNND	Mean nearest neighbor distance (the mean of the shortest distances connecting each species to any other species in the assemblage)	Webb, Ackerly, McPeek, and Donoghue (2002)
RaoQ	Quadratic entropy using plot‐specific trait values	Botta‐Dukat (2005)
FDis	Functional dispersion: weighted distances from a weighted centroid in multitrait space using plot‐specific trait values	Villeger et al. (2008)
*H′*	Shannon's diversity index	Tilman et al. (1996)

Note

The order from top to bottom for the measures of biodiversity represents their relative ranking using Akaike's information criterion weights.

Because of the strong correlation between most biodiversity indices (Spearman's ρ > 0.3; Table A3) and because multivariate functional indices are derived from the same trait data, we avoided including more than one of these like indices in any one model. Then, we constructed models with all remaining combinations of selected biodiversity metrics. At the meanwhile, we incorporated the interaction term between selected biodiversity metrics and experimental treatment into the model if multipliable model outperformed additive model considering treatment effects for particular selected biodiversity metrics. Finally, we also incorporated soil resources into above constructed models following the same constraint of correlation among soil resources and between selected biodiversity metrics and soil resources.

## 3 RESULTS

### 3.1 Comparisons between biodiversity metrics

As expected, phylogenetic and functional diversity indices alone outperformed traditional species richness and Shannon's evenness to explain the variation of biomass production when simultaneously considering elevation and treatment (Table 1, Table 2, Tables A2 and A4). Although only several functional diversity indices (*H*_max_, *RaoQ*,* FDis*,* FDiv*, detailed information see in Table A1) considering intraspecific variability attained greater model support than corresponding indices using species mean traits (Table 1, Table A2), most of these indices were selected as relatively better‐ranked single‐biodiversity metrics (Table 1). Of all functional diversity indices, the community‐level mean of “specific” maximum plant height (*H*_max_) on average accounted for the most explained variation in biomass production (*R*_m_^2^ > 50%; Table 1). Phylogenetic diversity (IAC) was the top‐ranked single‐biodiversity metric of all considered biodiversity metrics here and explained the most variation in biomass production (*R*_m_^2^ > 66%; Table A2).

**Table 2:** General linear mixed‐effect model (GLMM) results for biomass production as a function of several fixed factors and a hierarchical random factor

Model	LL	*k*	AIC_c_	ΔAIC_c_	*w*AIC_c_	*R*_m_^2^	*R*_c_^2^
*S* + IAC + *LN* + C + *T*	57.115	13	−85.145	0.000	0.291	91.8	99.9
*S* + IAC + *LN* + *T*	55.664	12	−84.706	0.439	0.234	91.6	99.9
*S* + IAC + *LN* + *N* + *T*	56.336	13	−83.588	1.557	0.134	91.7	99.9
*S* + IAC + *C* + *T*	54.529	12	−82.437	2.708	0.075	91.4	99.9
*S* + IAC + *T*	53.195	11	−82.189	2.955	0.066	91.3	99.9
*S* + IAC + FDis + *C* + *T*	55.010	13	−80.934	4.210	0.035	91.5	99.9
*S* + IAC + *N* + *T*	53.732	12	−80.843	4.302	0.034	91.3	99.9
*S* + IAC + FDis + *T*	53.725	12	−80.829	4.316	0.034	91.3	99.9
*S* + IAC + RaoQ + *C* + *T*	54.906	13	−80.726	4.418	0.032	91.5	99.9
*S* + IAC + RaoQ + *T*	53.642	12	−80.661	4.483	0.031	91.3	99.9

Notes

Fixed factors are number of species (*S*), Shannon's evenness (*H’*), and phylogenetic diversity (IAC, imbalance of abundance at the clade; MNND, mean nearest‐neighbor distance), and community‐level mean of single functional traits (*H*_max_, plot‐specific maximum plant height; *LN*, mean leaf nitrogen content value for individual species used for all plots where the species is found) or multivariate functional trait indices (RaoQ, Quadratic entropy; FDis, Functional dispersion: weighted distances from a weighted centroid in multitrait space), and experimental treatments (*T*:* T*_C_ = control, *T*_W_ = warming, *T*_N_ = nitrogen addition, *T*_WN_ = warming and nitrogen addition and *T*_G_ = livestock‐grazing), and soil resources (*C*, soil carbon content; *N*, soil total nitrogen content; *P*, soil total phosphorus content). Hierarchical random factor is elevation (2,700, 3,200, and 3,400 m), treatment, and plot. Values are shown for the estimated number of model parameters (*k*), maximum log‐likelihood (LL), and the information‐theoretic Akaike's information criterion corrected for small samples (AIC_c_), change in AIC_c_ relative to the top‐ranked model (ΔAIC_c_), AIC_c_ weight (*w*AIC_c_, model probability), and the marginal and total variance explained (*R*_m_^2^, *R*_c_^2^) as a measure of the model's goodness of fit. The top 10 models are listed; the full table is shown in Appendix: Table A3.

### 3.2 Biodiversity effects

**Figure 2:**
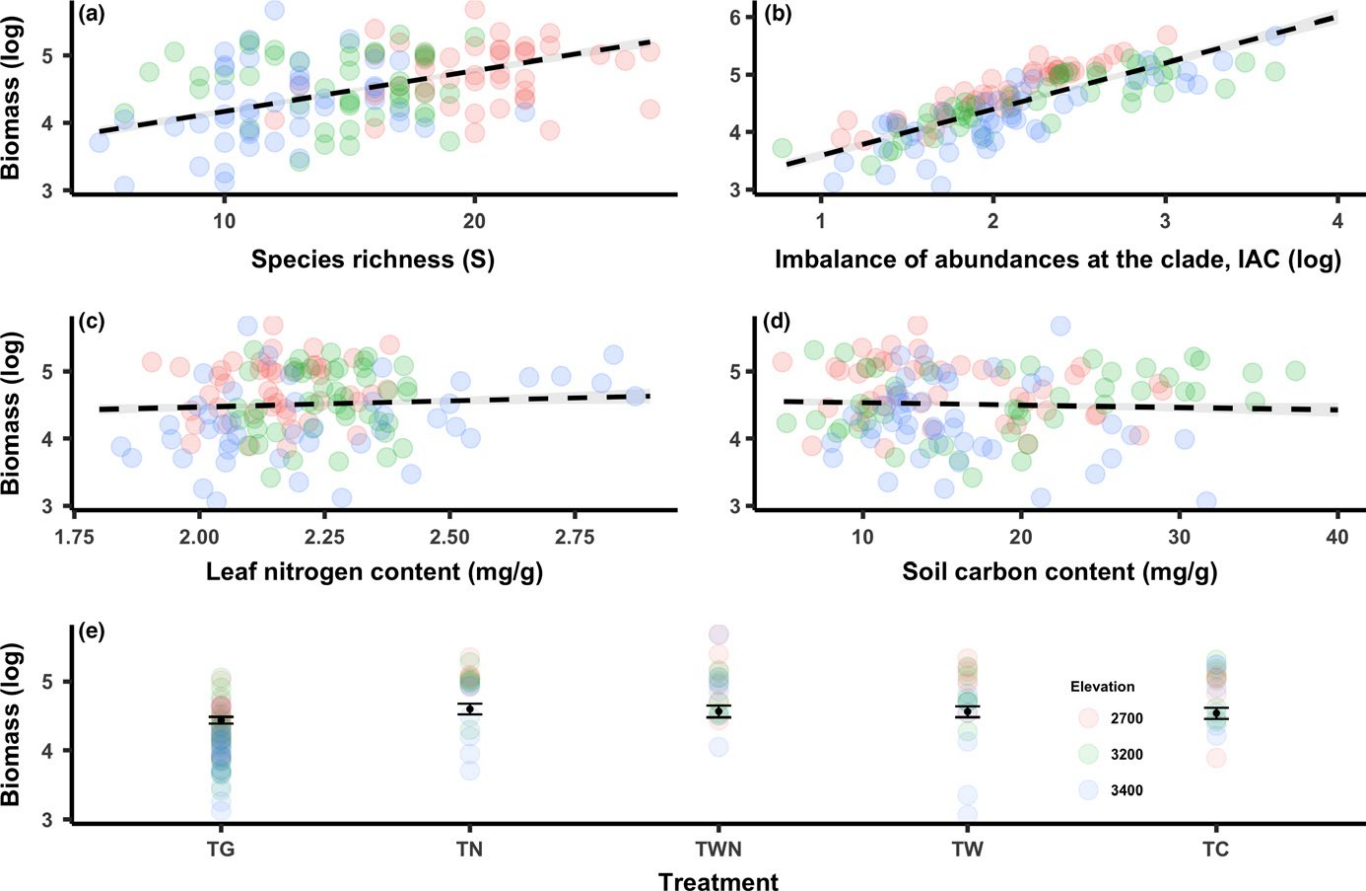
Scatter plots of the best‐supported variables combined in the general linear mixed‐effect models to predict variation in biomass production: (a) species richness (*S*), (b) imbalance of abundance at the clade (IAC) based on a maximum‐likelihood phylogeny, (c) community‐level mean of mean leaf nitrogen content value for individual species used for all plots where the species is found (LN), (d) soil carbon content (*C*), and (e) experimental treatments (*T*_C_ = control, *T*_W_ = warming, *T*_N_ = nitrogen addition, *T*_WN_ = warming and nitrogen addition, and *T*_G_ = livestock‐grazing). Dashed lines are linear regression lines, gray ribbon are their confidence intervals, and points and error bar in (e) are predicted values and their confidence intervals using general linear mixed‐effect model

Of the 166 multivariate linear mixed‐effect models, the most parsimonious model included species richness (*S*), phylogenetic diversity (IAC), the community‐level mean of “fixed” leaf nitrogen content (LN_*f*_), soil carbon content (C), and treatment (*T*) accounting for >91% of the deviance explained in productivity (Table 2). After accounting for confounding effects of environmental factors and experimental treatment, biomass production generally increased with increasing species richness, phylogenetic diversity, and the community‐level mean of “fixed” leaf nitrogen content (Figure 2a–c).

### 3.3 Environmental and treatment effects

We found relatively weaker environment and treatment effects on biomass production compared to those of selected biodiversity metrics, but few treatment effects on biodiversity–production relationship (Table 2, Table A2). The top‐ranked model showed that grazing strongly reduced the biomass production compared with nitrogen addition; however, nitrogen addition and experimental warming showed no impact on biomass production (Figure 2e). We also found evidence for a weak negative relationship between biomass production and soil carbon content (Figure 2d).

## 4 DISCUSSION

Our results show that phylogenetic and functional diversity alone outperformed traditional biodiversity measures, species richness, and Shannon's evenness, for explaining variation in productivity. This corroborates observational and experimental evidence that phylogenetic and functional measures better align with the mechanisms controlling community assembly and ecosystem function than taxonomic measures (Cadotte et al., 2009; Flynn et al., 2011; Liu, Zhang et al., 2015). Of all considered functional biodiversity indices, a single functional trait was the single best predictor of productivity patterns. This is not surprising since single functional trait might explain a larger amount of variation in productivity than multivariate functional indices likely due to functional trade‐offs and coordinated variation of functional traits (Cingolani, Cabido, Gurvich, Renison, & Diaz, 2007; Roscher et al., 2012).

Meanwhile, our study revealed that transitioning from using species mean (e.g., “fixed”) traits to plot level (e.g., “specific”) traits enhanced the explanatory power of functional diversity irrespective of plant traits in isolation or combination. Including specific traits allows us to detect subtle differences in functional diversity that respond to environmental variation that does not involve species turnover (Luo et al., 2016). Indeed, Jung et al. (2014) reported that the response of subalpine grassland communities to short extreme drought events was more mediated by intraspecific trait variability than species turnover. Intraspecific trait variability, through phenotypic plasticity, can promote species coexistence through providing fitness advantages and acting as a buffer against rapid climate change (Aspinwall et al., 2015; Nicotra et al., 2010; Valladares, Gianoli, & Gomez, 2007). This might lead to the shift in plant strategies in association with resource capture and use efficiencies at the local scale, which in turn are more related to plot‐specific aboveground biomass production. Furthermore, phenotypic plasticity, especially associating with maximum plant height, might ameliorate light competition, which is assumed to be an important mechanism explaining species loss and biodiversity effects (Borer et al., 2014; Cadotte, 2017; Fridley, 2003; Hautier, Niklaus, & Hector, 2009; Zhou et al., 2017). Our results generally supported these assumptions and highlighted the critical role of intraspecific trait variability in more precisely predicting the ecosystem functioning in the face of global climate change.

Although functional diversity could explain a substantial proportion of variation in productivity, the combination of phylogenetic diversity and a functional trait (leaf nitrogen) attained more model support and greater explanatory power. This implies that functional diversity and phylogenetic diversity could complement each other in the perspective of ecosystem functioning because of their own limitations. Functional diversity was limited by the absence of potential key functional traits, for example, belowground root traits in our study (Cadotte et al., 2009). Linkage between phylogenetic diversity and real ecological differences remains unclear (Cadotte, Davies, & Peres‐Neto, 2017). Thus, the influence of unmeasured plant traits might be compensated by metrics that capture phylogenetic information, such as the distribution of abundances at the clades or the equitability of abundance‐weighted entropic measure of the distribution of evolutionary distinctiveness in an assemblage (Cadotte et al., 2010). Such a combination of functional and phylogenetic information for explaining biodiversity–productivity relationships has received support from both biodiversity manipulation experiments and natural ecosystems (Liu, Zhang et al., 2015; Zhou et al., 2017). For example, Liu, Zhang et al. (2015) found that phylogenetic diversity and plant height represented the most parsimonious combination to predict aboveground biomass production in a removal experiment where species richness and functional diversity were manipulated in alpine meadows of the Tibetan Plateau.

In this study, we found strong and positive effects of species richness on productivity in natural ecosystems after accounting for potential confounding factors. This was consistent with a review by Tilman et al. (2014), in which the diversity effect is as great as, or greater than, the effects of herbivory, nitrogen addition, and other drivers of environmental change. Although our experiment is limited in the short term by the drivers of environmental change, our results still supported a strong positive species richness–productivity relationship in natural ecosystems even after quantifying the effects of intraspecific trait variability and evolutionary history. Despite our findings, the role of biodiversity in the productivity of natural ecosystem remains controversial (Adler et al., 2011), and our results emphasize the fact that we underestimate the importance of biodiversity for ecosystem function when we use species richness only.

Our results revealed that the drivers of environmental change had negligible effects on the relationship between biodiversity and aboveground biomass production. Our finding showed that the relationship between IAC and biomass production was consistently strongest for all considered biodiversity metrics in various treatments. Cadotte (2013) showed that biomass production was strongly predicted by phylogenetic diversity and that this finding might result from species complementarity, and ultimately species coexistence mechanisms (Chesson & Warner, 1981; Hodapp, Hillebrand, Blasius, & Ryabov, 2016; Horn & Macarthur, 1972; Levins & Culver, 1971). IAC that quantifies the relative deviation in the abundance distribution of a local community from a null distribution where individuals are evenly partitioned between clade splits can be used to infer the relative importance of competition and environmental filtering for local assembly. IAC would tend toward 0 if the strength of competition was proportional to phylogenetic relatedness, while IAC would be far greater than 0 if environmental filtering was key to community structure (Cadotte et al., 2010). Meanwhile, Cadotte (2017) showed that multidimensional trait measures might drive complementarity effect through niche complementarity, while few, singular traits (mainly height) might drive selection effect through interspecific competition. Our results were generally in line with these studies, because on the one hand, maximum plant height outperformed the multivariate functional indices alone in the perspective of ecosystem productivity, implying the importance of selection effect in biomass production in natural mountain grassland ecosystems; on the other hand, we observed IAC values far greater than 0, implying the dominance of environmental filtering in local community assembly, which might contribute to the role of selection effect in our system. Our results point to the importance of both complementarity effects and selection effects for aboveground biomass production in natural mountain grassland ecosystems.

## CONFLICT OF INTEREST

None declared.

## AUTHOR CONTRIBUTIONS

JJL conceived the idea and designed the experiment, JJL collected the data, JJL and MWC analyzed the data, JJL and MWC led the writing of the manuscript, and all authors contributed critically to the drafts and gave final approval for publication.

## DATA ACCESSIBILITY

Data available from the Dryad Digital Repository: https://doi.org/10.5061/dryad.5b02c11

## APPENDIX 1

### 1.1

**Table A1:** Measures of biodiversity considered in the analysis

Diversity measure	Description	References
*S*	Realized species richness of plot	Tilman et al. (1996)
*H′*	Shannon's diversity index	Tilman et al. (1996)
PD	Phylogenetic diversity (the sum of all phylogenetic branch lengths connecting species together)	Faith (1992)
MNND	Mean nearest neighbor distance (the mean of the shortest distances connecting each species to any other species in the assemblage)	Webb et al. (2002)
MPD	Mean pairwise distance (the mean of all the distances connecting species in an assemblage)	Webb et al. (2002)
MNND_ed_	Abundance‐weighted MNND	Webb et al. (2002)
MPD_ed_	Abundance‐weighted MPD	Webb et al. (2002)
E_AED_	Equitability of abundance‐weighted entropic measure of the distribution of evolutionary distinctiveness in an assemblage	Cadotte et al. (2010)
IAC	Imbalance of abundances among clades: measures the deviation in abundance distribution among internal splits from a null	Cadotte et al. (2010)
FEve	Functional Evenness: abundance‐weighted pairwise functional distances	Villeger et al. (2008)
FDis	Functional dispersion: weighted distances from a weighted centroid in multitrait space	Villeger et al. (2008)
FDiv	Functional divergence: mean abundance‐weighted deviance from an absolute abundance‐weighted deviance	Villeger et al. (2008)
FRic	Functional richness: convex hull volume of the trait space	Cornwell, Schwilk, & Ackerly, 2006; Villeger et al. (2008)
RaoQ	Quadratic entropy	Botta‐Dukat (2005)

**Table A2:** General linear mixed‐effect model (GLMM) results for biomass production as a function of single‐biodiversity metric, experimental treatment, and their interaction as fixed factor and hierarchical random factor

Model	LL	*k*	AIC_c_	ΔAIC_c_	*w*AIC_c_	*R*_m_^2^	*R*_c_^2^
IAC + *T*	−8.457	10	38.732	0.000	0.948	66.7	97.9
IAC × *T*	−6.473	14	44.535	5.803	0.052	67.7	99.9
*H*_max*s*_ + *T*	−44.775	10	111.367	72.635	<0.001	50.2	98.2
*H*_max*s*_ × *T*	−44.220	14	120.029	81.297	<0.001	50.4	99.9
LN_*f*_ + *T*	−63.666	10	149.151	110.419	<0.001	38.3	99.9
*S* + *T*	−63.748	10	149.315	110.583	<0.001	46.8	99.9
*S *× *T*	−58.939	14	149.468	110.735	<0.001	49.3	99.9
LN_*s*_ + *T*	−64.135	10	150.088	111.356	<0.001	37.8	99.9
NND + *T*	−65.273	10	152.364	113.632	<0.001	41.1	99.9
SLA_*f*_ + *T*	−65.969	10	153.755	115.023	<0.001	37.3	99.9
PD + *T*	−66.131	10	154.081	115.349	<0.001	43.4	99.9
PD × *T*	−61.507	14	154.603	115.871	<0.001	46.1	99.9
HEED + *T*	−67.131	10	156.080	117.348	<0.001	36.4	99.9
LN_*f*_ × *T*	−62.699	14	156.988	118.256	<0.001	38.9	99.9
LN_*s*_ × *T*	−62.708	14	157.006	118.274	<0.001	38.7	99.9
NRI_ed_ + *T*	−67.609	10	157.037	118.305	<0.001	35.2	99.9
RaoQ_*s*_ + *T*	−67.614	10	157.047	118.315	<0.001	36.3	99.9
SLA_*s*_ × *T*	−62.735	14	157.059	118.327	<0.001	42.1	99.9
FDis_*s*_ + *T*	−67.792	10	157.403	118.670	<0.001	36.5	99.9
LP_*s*_ + *T*	−67.829	10	157.477	118.744	<0.001	33.3	99.9
LP_*f*_ + *T*	−67.870	10	157.557	118.825	<0.001	33.3	99.9
*H′* × *T*	−63.169	14	157.927	119.195	<0.001	40.1	99.9
MPD + *T*	−68.332	10	158.483	119.751	<0.001	35.1	99.9
NND_ed_ + *T*	−68.340	10	158.498	119.765	<0.001	36.5	99.9
SLA_*f*_ × *T*	−63.507	14	158.604	119.872	<0.001	39.1	99.9
NND × *T*	−63.713	14	159.016	120.284	<0.001	41.9	99.9
*H′* + *T*	−68.852	10	159.523	120.79	<0.001	36.1	99.9
LC_*f*_ + *T*	−68.908	10	159.635	120.903	<0.001	35.1	99.9
MPD_ed_ × *T*	−64.030	14	159.650	120.918	<0.001	39.0	99.9
SLA_*s*_ + *T*	−69.078	10	159.975	121.243	<0.001	35.2	99.9
FDis_*f*_ + *T*	−69.096	10	160.010	121.278	<0.001	35.7	99.9
RaoQ_*f*_ + *T*	−69.164	10	160.146	121.414	<0.001	35.5	99.9
FRic_*f*_ + *T*	−69.264	10	160.347	121.615	<0.001	38.5	99.9
NRI + *T*	−69.288	10	160.393	121.661	<0.001	34.3	99.9
NTI + *T*	−69.311	10	160.441	121.709	<0.001	35.2	99.9
NTI_ed_ + *T*	−69.648	10	161.115	122.383	<0.001	34.7	99.9
FDiv_*s*_ + *T*	−69.710	10	161.238	122.506	<0.001	35.2	99.9
LP_*s*_ × *T*	−64.965	14	161.520	122.788	<0.001	35.1	99.9
LC_*s*_ + *T*	−69.879	10	161.576	122.844	<0.001	34.7	99.9
LP_*f*_ × *T*	−65.036	14	161.662	122.930	<0.001	35.1	99.9
*H*_max*f*_ + *T*	−70.006	10	161.830	123.098	<0.001	33.9	99.9
FRic_*s*_ × *T*	−65.158	14	161.906	123.173	<0.001	39.5	99.9
FDis_*s*_ × *T*	−65.229	14	162.047	123.315	<0.001	38.7	99.9
LC_*f*_ × *T*	−65.234	14	162.058	123.326	<0.001	38.5	99.9
NRI_ed_ × *T*	−65.310	14	162.210	123.478	<0.001	37.1	99.9
FRic_*s*_ + *T*	−70.254	10	162.327	123.595	<0.001	35.5	99.9
FEve_*f*_ + *T*	−70.337	10	162.492	123.760	<0.001	33.9	99.9
FDiv_*f*_ + *T*	−70.423	10	162.665	123.933	<0.001	34.4	99.9
HAED + *T*	−70.445	10	162.707	123.975	<0.001	33.9	99.9
FEve_*s*_ + *T*	−70.476	10	162.770	124.038	<0.001	33.9	99.9
MPD_ed_ + *T*	−70.476	10	162.771	124.039	<0.001	33.9	99.9
FRic_*f*_×*T*	−65.664	14	162.917	124.185	<0.001	41.1	99.9
NND_ed_×*T*	−65.853	14	163.296	124.564	<0.001	38.8	99.9
RaoQ_*s*_×*T*	−65.916	14	163.421	124.689	<0.001	37.8	99.9
MPD × *T*	−66.237	14	164.065	125.332	<0.001	36.9	99.9
HEED × *T*	−66.671	14	164.932	126.199	<0.001	36.5	99.9
NRI × *T*	−66.679	14	164.948	126.216	<0.001	36.5	99.9
NTI × *T*	−67.022	14	165.634	126.902	<0.001	37.4	99.9
FDiv_*s*_ × *T*	−67.470	14	166.530	127.798	<0.001	36.9	99.9
NTI_ed_ × *T*	−68.012	14	167.615	128.882	<0.001	35.8	99.9
FDis_*f*_ × *T*	−68.143	14	167.876	129.144	<0.001	36.5	99.9
RaoQ_*f*_ × *T*	−68.432	14	168.454	129.722	<0.001	36.0	99.9
*H*_max*f*_ × *T*	−68.722	14	169.034	130.302	<0.001	34.8	99.9
FEve_*f*_ × *T*	−69.058	14	169.706	130.974	<0.001	34.6	99.9
FEve_*s*_ × *T*	−69.170	14	169.930	131.198	<0.001	34.9	99.9
LC_*s*_ × *T*	−69.377	14	170.344	131.612	<0.001	35.0	99.9
HAED × *T*	−69.433	14	170.455	131.723	<0.001	34.4	99.9
FDiv_*f*_ × *T*	−69.783	14	171.155	132.423	<0.001	34.8	99.9
1	−80.940	5	172.356	133.624	<0.001	0.0	91.1

*Notes*

Fixed factors are number of species (*S*), Shannon's evenness (*H’*), and phylogenetic diversity (PD, sum of branch lengths; IAC, imbalance of abundance at the clade; EAED, equitability of abundance‐weighted entropic measure of the distribution of evolutionary distinctiveness; MPD, mean pairwise distance; MNND, mean nearest‐neighbor distance; MPD_ed_, weighted mean pairwise distance; MNND_ed_, weighted mean nearest neighbor distance); and community‐level mean of single functional traits using interspecific and intraspecific functional traits indicated by subscript “*f*” and “*s*,” respectively (*H*_max_ *,* maximum plant height*;* LC, leaf carbon content; LN, leaf nitrogen content; LP, leaf phosphorus content; SLA, specific leaf area) or multivariate functional trait indices using interspecific and intraspecific functional traits indicated by subscript “*f*” and “*s*,” respectively (FDis, functional distribution; FRic, functional richness; FEve, functional evenness; FDiv, functional divergence; RaoQ, quadratic entropy), and experimental treatments (*T*:* T*_C_ = control, *T*_W_ = warming, *T*_N_ = nitrogen addition, *T*_WN_ = warming and nitrogen addition, and *T*_G_ = livestock‐grazing). Hierarchical random factor is elevation (2,700, 3,200, and 3,400 m), treatment, and plot. Values are shown for the estimated number of model parameters (*k*), maximum log‐likelihood (LL), and the information‐theoretic Akaike's information criterion corrected for small samples (AIC_c_), change in AIC_c_ relative to the top‐ranked model (ΔAIC_c_), AIC_c_ weight (*w*AIC_c_, model probability), and the marginal and total variance explained (*R*_m_^2^, *R*_c_^2^) as a measure of the model's goodness of fit.

**Table A3:** Spearman's ρ correlation matrix for raw input variables

	*S*	*H’*	PD	IAC	HEED	HAED	MPD	MPD_ed_	MNND	MNND_ed_	FRic_*f*_	FEve_*f*_	FDiv_*f*_	FDisf	RaoQ_*f*_	*H*_max*f*_	*LC*_*f*_	*LN*_*f*_	*LP*_*f*_	SLA_*f*_	FRic_*s*_	FEve_*s*_	FDivs	FDiss	RaoQs	Hmaxs	LCs	LNs	LPs	SLAs	pH	P	N
*H’*	0.65																																
PD	0.94	0.60																															
IAC	−0.22	−0.41	−0.18																														
HEED	0.16	0.31	0.09	−0.42																													
HAED	0.27	0.35	0.20	−0.23	0.96																												
MPD	−0.03	−0.18	0.15	0.13	−0.20	−0.17																											
MPD_ed_	0.41	0.72	0.44	−0.37	0.20	0.23	0.18																										
MNND	−0.62	−0.45	−0.43	0.18	−0.26	−0.33	0.39	−0.22																									
MNND_ed_	−0.33	−0.11	−0.24	−0.05	−0.04	−0.07	0.23	0.23	0.54																								
FRic_*f*_	0.85	0.44	0.81	−0.12	0.09	0.19	0.12	0.33	−0.49	−0.27																							
FEve_*f*_	−0.27	−0.14	−0.26	0.02	0.08	0.03	−0.15	−0.25	0.10	0.06	−0.29																						
FDiv_*f*_	0.26	0.02	0.27	0.12	0.00	0.08	0.16	0.06	−0.15	−0.11	0.37	0.05																					
FDis_*f*_	0.27	0.20	0.21	0.07	0.04	0.11	−0.08	0.00	−0.25	−0.16	0.31	0.22	0.64																				
RaoQ_*f*_	0.24	0.21	0.18	0.06	0.05	0.11	−0.12	−0.03	−0.24	−0.13	0.24	0.27	0.59	0.96																			
*H*_max*f*_	−0.44	−0.31	−0.40	0.45	−0.16	−0.13	−0.01	−0.22	0.33	0.17	−0.34	0.28	0.07	0.06	0.05																		
*LC*_*f*_	−0.06	−0.07	−0.04	−0.12	−0.05	−0.07	0.22	0.13	0.02	0.10	0.00	−0.20	0.01	−0.15	−0.16	−0.27																	
*LN*_*f*_	−0.07	0.11	−0.10	0.18	0.05	0.08	−0.36	−0.16	0.05	0.17	−0.22	0.35	−0.04	0.21	0.28	0.34	−0.27																
*LP*_*f*_	−0.44	−0.11	−0.45	0.07	−0.06	−0.13	−0.35	−0.27	0.31	0.25	−0.54	0.37	−0.30	−0.02	0.10	0.40	−0.29	0.54															
*SLA*_*f*_	−0.30	−0.33	−0.22	0.14	−0.10	−0.12	0.49	−0.07	0.29	−0.04	−0.12	−0.12	−0.06	−0.26	−0.33	0.23	0.29	−0.50	−0.37														
FRic_*s*_	0.86	0.46	0.81	−0.17	0.08	0.18	0.12	0.35	−0.48	−0.27	0.96	−0.28	0.35	0.31	0.25	−0.38	0.00	−0.25	−0.52	−0.15													
FEve_*s*_	−0.29	−0.16	−0.26	0.02	0.04	−0.04	−0.17	−0.24	0.18	0.10	−0.36	0.77	0.03	0.17	0.22	0.27	−0.15	0.33	0.44	−0.07	−0.34												
FDiv_*s*_	0.25	0.09	0.25	0.08	−0.02	0.06	0.07	0.11	−0.18	0.00	0.32	0.13	0.80	0.62	0.59	0.12	0.00	0.06	−0.10	−0.19	0.30	0.10											
FDis_*s*_	0.24	0.23	0.21	0.05	0.02	0.06	−0.10	0.01	−0.24	−0.07	0.22	0.33	0.45	0.80	0.78	0.08	−0.18	0.28	0.11	−0.24	0.22	0.30	0.64										
RaoQ_*s*_	0.20	0.23	0.17	0.06	0.02	0.07	−0.13	−0.03	−0.20	−0.05	0.15	0.37	0.39	0.73	0.77	0.10	−0.20	0.34	0.22	−0.29	0.15	0.35	0.57	0.96									
*H*_max*s*_	−0.18	−0.11	−0.14	0.70	−0.19	−0.03	0.03	−0.11	0.15	0.08	−0.09	0.11	0.07	0.08	0.06	0.68	−0.19	0.28	0.22	0.08	−0.15	0.11	0.12	0.13	0.16								
*LC*_*s*_	−0.09	−0.02	−0.08	−0.12	0.05	0.00	0.05	0.07	0.02	0.02	−0.11	0.05	−0.17	−0.22	−0.23	−0.21	0.69	−0.12	−0.07	0.16	−0.11	0.05	−0.15	−0.05	−0.02	−0.12							
*LN*_*s*_	−0.08	0.12	−0.11	0.16	0.08	0.10	−0.36	−0.15	0.05	0.14	−0.24	0.39	−0.09	0.16	0.23	0.31	−0.25	0.97	0.55	−0.48	−0.27	0.36	0.01	0.29	0.37	0.27	0.06						
*LP*_*s*_	−0.44	−0.11	−0.45	0.07	−0.06	−0.13	−0.35	−0.27	0.30	0.24	−0.54	0.37	−0.31	−0.03	0.08	0.40	−0.29	0.54	1.00	−0.37	−0.52	0.45	−0.11	0.11	0.23	0.22	−0.03	0.56					
*SLA*_*s*_	−0.14	0.03	−0.07	−0.05	−0.01	−0.03	0.33	0.18	0.15	−0.02	−0.02	−0.21	−0.07	−0.17	−0.22	−0.09	0.25	−0.46	−0.31	0.64	−0.03	−0.24	−0.09	−0.08	−0.11	−0.09	0.10	−0.46	−0.32				
pH	0.70	0.36	0.71	−0.06	0.11	0.22	0.16	0.30	−0.38	−0.15	0.71	−0.31	0.32	0.19	0.14	−0.41	0.08	−0.17	−0.63	−0.06	0.70	−0.39	0.28	0.15	0.07	−0.12	−0.08	−0.20	−0.64	0.05			
*P*	−0.58	−0.17	−0.59	0.05	−0.07	−0.16	−0.21	−0.26	0.36	0.21	−0.64	0.29	−0.39	−0.16	−0.07	0.28	−0.13	0.22	0.72	−0.07	−0.62	0.31	−0.25	−0.02	0.08	0.14	0.12	0.27	0.73	−0.05	−0.75		
*N*	−0.24	−0.15	−0.25	0.21	−0.22	−0.20	0.06	−0.15	0.17	−0.09	−0.14	0.09	−0.01	0.01	0.01	0.18	0.00	0.05	0.02	0.17	−0.12	0.06	−0.05	0.00	0.01	0.22	0.05	0.06	0.03	0.07	−0.35	0.27	
*C*	−0.23	−0.20	−0.24	0.17	−0.18	−0.18	0.13	−0.10	0.17	−0.05	−0.10	0.06	−0.08	−0.06	−0.09	0.17	−0.02	0.03	0.01	0.14	−0.09	0.05	−0.08	−0.08	−0.08	0.18	0.03	0.04	0.01	0.00	−0.31	0.15	0.74

*Notes*

Shown are species richness (*S*), Shannon's evenness (*H’*), phylogenetic diversity (PD, sum of branch lengths; IAC, imbalance of abundance at the clade; EAED, equitability of abundance‐weighted entropic measure of the distribution of evolutionary distinctiveness; MPD, mean pairwise distance; MNND, mean nearest neighbor distance; MPD_ed_, weighted mean pairwise distance; MNND_ed_, weighted mean nearest neighbor distance); multivariate functional trait indices based on interspecific and intraspecific functional traits indicated by subscript “f” and “s,” respectively (FDis, functional distribution; FRic, functional richness; FEve, functional evenness; FDiv, functional divergence; RaoQ, quadratic entropy), community‐level mean of single functional traits also using interspecific and intraspecific functional traits indicated by subscript “f” and “s,” respectively (*H*_max_, maximum plant height; *LC*, leaf carbon content; *LN*, leaf nitrogen content; *LP*, leaf phosphorus content; *SLA*, specific leaf area).

**Table A4:** General linear mixed‐effect model (GLMM) results for biomass production as a function of several fixed factors and a hierarchical random factor

Model	LL	*k*	AIC_c_	ΔAIC_c_	*w*AIC_c_	*R*_m_^2^	*R*_c_^2^
*S* + IAC + *LN* + *C* + *T*	57.115	13	−85.145	0.000	0.291	91.8	99.9
*S* + IAC + *LN* + *T*	55.664	12	−84.706	0.439	0.234	91.6	99.9
*S* + IAC + *LN* + *N* + *T*	56.336	13	−83.588	1.557	0.134	91.7	99.9
*S* + IAC + *C* + *T*	54.529	12	−82.437	2.708	0.075	91.4	99.9
*S* + IAC + *T*	53.195	11	−82.189	2.955	0.066	91.3	99.9
*S* + IAC + FDis + *C* + *T*	55.010	13	−80.934	4.210	0.035	91.5	99.9
*S* + IAC + *N* + *T*	53.732	12	−80.843	4.302	0.034	91.3	99.9
*S* + IAC + FDis + *T*	53.725	12	−80.829	4.316	0.034	91.3	99.9
*S* + IAC + RaoQ + *C* + *T*	54.906	13	−80.726	4.418	0.032	91.5	99.9
*S* + IAC + RaoQ + *T*	53.642	12	−80.661	4.483	0.031	91.3	99.9
*S* + IAC + FDis + *N* + *T*	54.336	13	−79.588	5.556	0.018	91.4	99.9
*S* + IAC + RaoQ + *N* + *T*	54.255	13	−79.425	5.719	0.017	91.4	99.9
IAC + NND + *LN* + *C* + *T*	20.838	13	−12.592	72.553	<0.001	78.2	98.3
IAC + NND + *LN* + *N* + *T*	19.234	13	−9.383	75.762	<0.001	77.9	98.2
IAC + NND + *LN* + *T*	17.491	12	−8.360	76.785	<0.001	77.1	98.2
IAC + NND + RaoQ + *C* + *T*	17.236	13	−5.386	79.758	<0.001	78.5	98.4
IAC + NND + FDis + *C* + *T*	16.871	13	−4.657	80.487	<0.001	78.8	98.3
IAC + NND + RaoQ + *N* + *T*	15.996	13	−2.907	82.238	<0.001	78.5	98.3
IAC + NND + *C* + *T*	14.646	12	−2.670	82.475	<0.001	78.3	98.1
IAC + NND + FDis + *N* + *T*	15.593	13	−2.101	83.043	<0.001	78.7	98.2
IAC + NND + RaoQ + *T*	14.180	12	−1.737	83.407	<0.001	77.6	98.1
IAC + NND + FDis + *T*	13.832	12	−1.042	84.102	<0.001	77.9	98.1
IAC + NND + *N* + *T*	13.113	12	0.396	85.541	<0.001	78.0	98.1
IAC + NND + *T*	11.641	11	0.918	86.062	<0.001	77.2	98.0
IAC + *LN* + pH + *T*	6.846	12	12.929	98.074	<0.001	82.3	99.9
IAC + RaoQ + pH + *T*	6.242	12	14.139	99.283	<0.001	81.8	99.9
IAC + FDis + pH + *T*	5.845	12	14.932	99.9.077	<0.001	82.1	99.9
IAC + *LN* + *C* + *T*	2.756	12	21.109	106.254	<0.001	69.0	99.9
IAC + pH + *T*	1.462	11	21.276	106.420	<0.001	80.8	99.9
IAC + RaoQ + *C* + *T*	2.402	12	21.818	106.962	<0.001	70.3	99.9
IAC + FDis + *C* + *T*	1.676	12	23.270	108.414	<0.001	70.6	99.9
IAC + *LN* + *P* + *C* + *T*	2.859	13	23.367	108.511	<0.001	67.5	99.9
IAC + RaoQ + *P* + *C* + *T*	2.428	13	24.229	109.373	<0.001	69.5	99.9
IAC + RaoQ + *N* + *T*	0.789	12	25.045	110.189	<0.001	70.2	99.9
IAC + FDis + *P* + *C* + *T*	1.745	13	25.594	110.739	<0.001	69.3	99.9
IAC + *LN* + *N* + *T*	0.252	12	26.118	111.262	<0.001	68.7	99.1
IAC + FDis + *N* + *T*	−0.088	12	26.797	111.942	<0.001	70.6	99.9
IAC + RaoQ + *P* + *N* + *T*	0.794	13	27.497	112.642	<0.001	69.9	99.9
IAC + RaoQ + *T*	−1.812	11	27.824	112.968	<0.001	68.9	99.9
IAC + *LN* + *P* + *N* + *T*	0.308	13	28.468	113.613	<0.001	67.3	98.6
IAC + *LN* + *T*	−2.241	11	28.682	113.827	<0.001	67.1	98.1
IAC + FDis + *P* + *N* + *T*	−0.058	13	29.200	114.345	<0.001	69.7	99.9
IAC + FDis + *T*	−2.728	11	29.656	114.800	<0.001	69.2	99.9
IAC + RaoQ + *P* + *T*	−1.670	12	29.961	115.106	<0.001	70.8	99.9
IAC + *LN* + *P* + *T*	−2.219	12	31.059	116.204	<0.001	68.0	98.3
IAC + *C* + *T*	−3.686	11	31.572	116.716	<0.001	68.6	99.9
IAC + FDis + *P* + *T*	−2.648	12	31.919	117.063	<0.001	70.7	99.9
IAC + *P* + *C* + *T*	−3.352	12	33.327	118.471	<0.001	65.7	99.9
IAC + *N* + *T*	−6.157	11	36.515	121.659	<0.001	68.2	99.9
IAC + *P* + *N* + *T*	−5.938	12	38.497	123.642	<0.001	65.7	98.6
IAC + *T*	−8.457	10	38.732	123.877	<0.001	66.7	97.9
IAC + *P* + *T*	−8.445	11	41.091	126.235	<0.001	66.1	97.9
*H*_max_ + *LN* + pH + *T*	−36.620	12	99.861	185.006	<0.001	65.3	99.9
*H*_max_ + pH + *T*	−38.365	11	99.9.930	186.075	<0.001	64.4	99.9
*S* + *H*_max_ + *T*	−38.574	11	101.347	186.492	<0.001	61.4	95.9
*S* + *H*_max_ + *LN* + *T*	−37.695	12	102.011	187.156	<0.001	60.4	96.4
*S* + *H*_max_ + *C* + *T*	−37.828	12	102.278	187.422	<0.001	61.2	96.0
*S* + *H*_max_ + *LN* + *C* + *T*	−36.857	13	102.798	187.943	<0.001	60.3	96.3
*S* + *H*_max_ + *N* + *T*	−38.205	12	103.032	188.177	<0.001	61.5	95.9
*S* + *H*_max_ + *LN* + *N* + *T*	−37.276	13	103.637	188.782	<0.001	60.4	96.5
NND + *H*_max_ + *LN* + *C* + *T*	−37.453	13	103.991	189.136	<0.001	56.3	96.3
NND + *H*_max_ + *LN* + *T*	−38.868	12	104.358	189.502	<0.001	55.6	96.3
NND + *H*_max_ + *C* + *T*	−39.270	12	105.162	190.307	<0.001	56.4	96.0
NND + *H*_max_ + *LN* + *N* + *T*	−38.097	13	105.278	190.422	<0.001	56.2	96.4
NND + *H*_max_ + *T*	−40.642	11	105.484	190.629	<0.001	55.7	95.9
NND + *H*_max_ + *N* + *T*	−39.902	12	106.425	191.570	<0.001	56.2	96.0
*H*_max_ + *LN* + *C* + *T*	−40.844	12	108.309	193.454	<0.001	51.6	99.9
*H*_max_ + *LN* + *T*	−42.684	11	109.567	194.712	<0.001	50.7	99.9
*H*_max_ + *LN* + *N* + *T*	−41.609	12	109.840	194.985	<0.001	51.5	99.9
*H*_max_ + *C* + *T*	−42.933	11	110.066	195.211	<0.001	51.3	97.2
*H’* + *H*_max_ + *LN* + *C* + *T*	−40.551	13	110.187	195.332	<0.001	51.3	99.9
*H*_max_ + *LN* + *P* + *C* + *T*	−40.686	13	110.458	195.602	<0.001	53.8	99.9
*H*_max_ + *LN* + *P* + *T*	−42.179	12	110.980	196.125	<0.001	54.6	99.9
*H*_max_ + *T*	−44.775	10	111.367	196.512	<0.001	50.2	98.2
*H*_max_ + *N* + *T*	−43.679	11	111.558	196.702	<0.001	51.1	99.9
H + *H*_max_ + *LN* + *T*	−42.557	12	111.736	196.881	<0.001	50.4	99.9
*H’* + *H*_max_ + *LN* + *N* + *T*	−41.403	13	111.891	197.036	<0.001	51.3	99.9
*H*_max_ + *LN* + *P* + *N* + *T*	−41.410	13	111.905	197.050	<0.001	54.0	99.9
*H*_max_ + *P* + *C* + *T*	−42.856	12	112.334	197.478	<0.001	52.9	96.9
*H’* + *H*_max_ + *C* + *T*	−42.865	12	112.352	197.497	<0.001	51.1	97.5
*H’* + *H*_max_ + *LN* + *P* + *C* + *T*	−40.477	14	112.544	197.688	<0.001	52.8	99.9
*H*_max_ + *P* + *T*	−44.432	11	113.065	198.209	<0.001	53.6	97.1
*H’* + *H*_max_ + *LN* + *P* + *T*	−42.136	13	113.357	198.501	<0.001	54.1	99.9
*H’* + *H*_max_ + *T*	−44.766	11	113.733	198.877	<0.001	50.1	98.3
*H*_max_ + *P* + *N* + *T*	−43.586	12	113.793	198.938	<0.001	52.8	98.4
*H’* + *H*_max_ + *N* + *T*	−43.643	12	113.907	199.052	<0.001	50.9	99.9
*H’* + *H*_max_ + *LN* + *P* + *N* + *T*	−41.283	14	114.157	199.301	<0.001	53.2	99.9
*H’* + *H*_max_ + *P* + *C* + *T*	−42.821	13	114.727	199.871	<0.001	52.4	97.2
*H’* + *H*_max_ + *P* + *T*	−44.429	12	115.480	200.624	<0.001	53.8	97.1
*H’* + *H*_max_ + *P* + *N* + *T*	−43.573	13	116.231	201.375	<0.001	52.5	99.9
*LN* + pH + *T*	−57.526	11	139.252	224.396	<0.001	52.2	99.9
NND + *LN* + *T*	−58.903	11	142.006	227.150	<0.001	44.3	99.9
*S* + *LN* + *T*	−58.913	11	142.027	227.171	<0.001	48.2	99.9
NND + *LN* + *C* + *T*	−57.908	12	142.439	227.583	<0.001	44.9	97.7
*S* + *LN* + *C* + *T*	−58.424	12	143.471	228.615	<0.001	47.9	99.9
NND + *LN* + *N* + *T*	−58.753	12	144.128	229.273	<0.001	44.5	99.9
*S* + *LN* + *N* + *T*	−58.896	12	144.414	229.559	<0.001	48.2	99.9
*LN* + *C* + *T*	−62.205	11	148.611	233.755	<0.001	39.4	99.9
*LN* + *T*	−63.666	10	149.151	234.296	<0.001	38.3	99.9
RaoQ + pH + *T*	−62.497	11	149.193	234.338	<0.001	48.4	99.9
*S* + *T*	−63.748	10	149.315	234.459	<0.001	46.8	99.9
FDis + pH + *T*	−62.610	11	149.420	234.565	<0.001	48.5	99.9
*H’* + *LN* + *P* + *T*	−61.491	12	149.605	234.749	<0.001	50.1	99.9
*S* + FDis + *T*	−62.862	11	149.923	235.068	<0.001	46.9	99.9
*LN* + *P* + *T*	−62.889	11	149.979	235.123	<0.001	49.1	99.9
*S* + RaoQ + *T*	−62.967	11	150.134	235.278	<0.001	46.6	99.9
*H’* + *LN* + *C* + *T*	−61.896	12	150.414	235.558	<0.001	40.0	99.9
*H’* + *LN* + *T*	−63.172	11	150.544	235.689	<0.001	39.1	99.9
*LN* + *P* + *C* + *T*	−62.024	12	150.671	235.815	<0.001	41.8	99.9
pH + *T*	−64.576	10	150.970	236.114	<0.001	47.1	99.9
*LN* + *N* + *T*	−63.394	11	150.988	236.133	<0.001	38.7	99.9
*S* + *C* + *T*	−63.454	11	151.108	236.253	<0.001	46.6	99.9
*H’* + *LN* + *P* + *C* + *T*	−61.099	13	151.283	236.427	<0.001	50.4	99.9
*S* + *N* + *T*	−63.748	11	151.696	236.84	<0.001	46.8	99.9
*S* + FDis + *C* + *T*	−62.554	12	151.730	236.875	<0.001	46.7	99.9
NND + RaoQ + *T*	−63.844	11	151.888	237.033	<0.001	41.7	99.9
*S* + RaoQ + *C* + *T*	−62.660	12	151.942	237.087	<0.001	46.3	99.9
NND + FDis + *T*	−63.922	11	152.045	237.189	<0.001	42.0	99.9
*H’* + *LN* + *P* + *N* + *T*	−61.490	13	152.064	237.209	<0.001	50.1	99.9
*S* + FDis + *N* + *T*	−62.854	12	152.330	237.475	<0.001	46.9	99.9
NND + *T*	−65.273	10	152.364	237.509	<0.001	41.1	99.9
*LN* + *P* + *N* + *T*	−62.874	12	152.369	237.513	<0.001	49.1	99.9
*H’* + *LN* + *N* + *T*	−62.944	12	152.510	237.654	<0.001	39.4	99.9
*S* + RaoQ + *N* + *T*	−62.960	12	152.542	237.687	<0.001	46.6	99.9
NND + RaoQ + *C* + *T*	−63.067	12	152.757	237.901	<0.001	42.2	99.9
NND + FDis + *C* + *T*	−63.112	12	152.845	237.99	<0.001	42.5	99.9
NND + *C* + *T*	−64.367	11	152.934	238.078	<0.001	41.6	99.9
NND + RaoQ + *N* + *T*	−63.714	12	154.050	239.194	<0.001	41.9	99.9
NND + FDis + *N* + *T*	−63.784	12	154.190	239.334	<0.001	42.2	99.9
NND + *N* + *T*	−65.159	11	154.519	239.663	<0.001	41.3	99.9
*H’* + RaoQ + *P* + *T*	−65.054	12	156.730	241.874	<0.001	47.4	99.9
RaoQ + *P* + *T*	−66.341	11	156.882	242.027	<0.001	46.3	99.9
*H’* + FDis + *P* + *T*	−65.162	12	156.947	242.091	<0.001	47.3	99.9
RaoQ + *T*	−67.614	10	157.047	242.192	<0.001	36.3	99.9
RaoQ + *C* + *T*	−66.522	11	157.245	242.389	<0.001	37.2	99.9
FDis + *P* + *T*	−66.568	11	157.336	242.481	<0.001	46.1	99.9
FDis + *T*	−67.792	10	157.403	242.547	<0.001	36.5	99.9
FDis + *C* + *T*	−66.634	11	157.469	242.613	<0.001	37.4	99.9
*H’* + RaoQ + *T*	−66.890	11	157.980	243.125	<0.001	37.4	99.9
*H’* + FDis + *T*	−66.955	11	158.109	243.254	<0.001	37.7	99.9
*H’* + RaoQ + *C* + *T*	−65.963	12	158.549	243.693	<0.001	38.1	99.9
*H’* + FDis + *C* + *T*	−65.991	12	158.603	243.747	<0.001	38.4	99.9
RaoQ + *P* + *C* + *T*	−66.035	12	158.693	243.837	<0.001	46.6	99.9
*H’* + RaoQ + *P* + *C* + *T*	−64.863	13	158.812	243.956	<0.001	47.5	99.9
*H’* + *P* + *T*	−67.316	11	158.831	243.976	<0.001	45.5	99.9
RaoQ + *N* + *T*	−67.379	11	158.958	244.103	<0.001	36.6	99.9
*H’* + FDis + *P* + *C* + *T*	−64.937	13	158.959	244.104	<0.001	47.5	99.9
FDis + *P* + *C* + *T*	−66.201	12	159.024	244.169	<0.001	46.4	99.9
*H’* + RaoQ + *P* + *N* + *T*	−65.053	13	159.191	244.336	<0.001	47.4	99.9
FDis + *N* + *T*	−67.540	11	159.280	244.425	<0.001	36.8	99.9
RaoQ + *P* + *N* + *T*	−66.331	12	159.283	244.427	<0.001	46.3	99.9
*H’* + FDis + *P* + *N* + *T*	−65.159	13	159.404	244.548	<0.001	47.3	99.9
*H’* + *T*	−68.852	10	159.523	244.667	<0.001	36.1	99.9
FDis + *P* + *N* + *T*	−66.548	12	159.719	244.863	<0.001	46.2	99.9
*H’* + *C* + *T*	−67.783	11	159.765	244.91	<0.001	37.0	99.9
*H’* + RaoQ + *N* + *T*	−66.703	12	160.028	245.172	<0.001	37.7	99.9
*C* + *T*	−69.120	10	160.058	245.203	<0.001	35.1	99.9
*H’* + FDis + *N* + *T*	−66.758	12	160.137	245.281	<0.001	38.0	99.9
*H’* + *P* + *C* + *T*	−67.013	12	160.648	245.792	<0.001	45.8	99.9
*H’* + *P* + *N* + *T*	−67.315	12	161.252	246.396	<0.001	45.5	99.9
*H’* + *N* + *T*	−68.691	11	161.582	246.727	<0.001	36.4	99.9
*P* + *T*	−69.946	10	161.710	246.855	<0.001	43.3	99.9
*P* + *C* + *T*	−69.089	11	162.377	247.522	<0.001	36.2	99.9
*N* + *T*	−70.296	10	162.410	247.555	<0.001	34.3	99.9
*P* + *N* + *T*	−69.926	11	164.052	249.197	<0.001	43.3	99.9
1	−80.940	5	172.356	257.501	<0.001	0.0	91.1

*Notes*

Fixed factors are number of species (*S*), Shannon's evenness (*H’*), and phylogenetic diversity (IAC, imbalance of abundance at the clade; MNND, mean nearest neighbor distance), and community‐level mean of single functional traits (*H*_max_, plot‐specific maximum plant height; *LN*, mean leaf nitrogen content value for individual species used for all plots where the species is found) or multivariate functional trait indices (RaoQ, Quadratic entropy; FDis, Functional dispersion: weighted distances from a weighted centroid in multitrait space), and experimental treatments (*T*:* T*_C_ = control, *T*_W_ = warming, *T*_N_ = nitrogen addition, *T*_WN_ = warming and nitrogen addition, and *T*_G_ = livestock‐grazing), and soil resources (*C*, soil carbon content; *N*, soil total nitrogen content; *P*, soil total phosphorus content). Hierarchical random factor is elevation (2700 m, 3200 m and 3400 m), treatment, and plot. Values are shown for the estimated number of model parameters (*k*), maximum log‐likelihood (LL), and the information‐theoretic Akaike's information criterion corrected for small samples (AIC_c_), change in AIC_c_ relative to the top‐ranked model (ΔAIC_c_), AIC_c_ weight (*w*AIC_c_, model probability), and the marginal and total variance explained (R_m_^2^, R_c_^2^) as a measure of the model's goodness of fit.
